# Changes in hemoglobin oxidation and band 3 during blood storage impact oxygen sensing and mitochondrial bioenergetic pathways in the human pulmonary arterial endothelial cell model

**DOI:** 10.3389/fphys.2023.1278763

**Published:** 2023-10-16

**Authors:** Sirsendu Jana, Tigist Kassa, Francine Wood, Wayne Hicks, Abdu I. Alayash

**Affiliations:** Laboratory of Biochemistry and Vascular Biology, Center for Biologics Evaluation and Research Food and Drug Administration (FDA), Silver Spring, MD, United States

**Keywords:** aged RBC, oxidation, hemoglobin, pulmonary endothelial cells, band 3

## Abstract

Red blood cells (RBCs) undergo metabolic, oxidative, and physiological changes during storage, collectively described as the “storage lesion.” The impact of storage on oxygen homeostasis, following transfusion, is not fully understood. We show that RBC storage induces changes in oxygen binding that were linked to changes in oxygen sensing (hypoxia-inducible factor, HIF-1α) mechanisms and mitochondrial respiration in human pulmonary arterial endothelial cells (HPAECs). A decrease in oxygen affinity (P_50_) to approximately 20 from 30 mmHg was seen at the first week but remained unchanged for up to 42 days. This led to the suppression of HIF-1α in the first 3 weeks due to limited oxygen supplies by RBCs. Furthermore, membrane oxidative damage, band 3 alterations, and subsequent microparticle (MP) formation were also noted. Mass spectrometric analysis revealed the upregulation of transitional endoplasmic reticulum ATPase, essential for clearing ROS-damaged membrane proteins and the protein DDI1 homolog, a proteasomal shuttle chaperone. Band 3 complex proteins and superoxide dismutase were among the downregulated proteins. Mitochondrial oxygen consumption rates measured in HPAECs incubated with RBC-derived MPs (14-day and 42-day) showed a rise in maximal respiration. Intervention strategies that target intracellular hemoglobin (Hb)’s redox transitions and membrane changes may lead to the reestablishment of oxygen homeostasis in old RBCs.

## Introduction

Overall, blood supplies in the United States are considered safe, although concerns still remain about the biochemical changes that are known to occur during storage. The contamination of blood with pathogens and the methods used to inactivate blood have also been the subject of concerns as these technologies can compromise the safety of donated blood (Seltsam, 2017).

RBCs are the most frequently transfused blood product, with approximately 11 million red blood cells being transfused annually as part of the standard practices to increase the oxygen-carrying capacity and RBC mass (Hess, 2014; Storch et al., 2019). The storage of RBCs results in physiologic changes, including impaired nitric oxide (NO) metabolism; the accumulation of lactic acid; decreased pH, ATP, and 2,3-diphosphoglycerate (2,3-DPG); the release of inflammatory mediators; and increased red blood cell membrane inflexibility (Wolfe, 1985; Yoshida et al., 2019). Collectively, these metabolic changes are known as the “storage lesion,” which may contribute to impaired oxygen uptake, but clinical trials have produced conflicting results (D'Alessandro et al., 2010). A recent retrospective study showed that the transfusion of RBCs older than 35 days was associated with an increased length of stay in the hospital, morbidity, and mortality, especially for high-risk patients. However, on the other hand, several randomized trials suggested that patients transfused with short- or long-term stored RBCs have similar clinical outcomes (Klein, 2017).

RBCs are obtained either from donated whole-blood or collected by apheresis technology with the volume of an average unit of packed RBCs ranging from 200 to 350 mL with a high hematocrit level. The shelf life of RBCs varies depending on the anticoagulant preservative used in bags. Generally, in the United States, RBC units in additive solutions can be stored for up to 42 days at 4°C–6°C. One unit of blood is expected to raise the Hb concentration of an adult patient by 1 g/dL or hematocrit by approximately 3% (Hess and Greenwalt, 2002).

During prolonged storage, several subtle and not-so-subtle metabolic and structural changes occur within RBCs. This includes membrane vesiculation, microparticle (MP) formation, Hb’s oxidative modifications, and the progressive failure of cellular homeostasis and antioxidant defenses (Said et al., 2017). Post-translational modifications (PTMs), such as phosphorylation, oxidation, and aggregation, are functionally involved in the regulation of RBC homeostasis and lifespan. Storage lesions are also affected by internal redox oxidative reactions; hence, it is currently recognized as an “oxidative lesion” (Alayash, 2022).

Under physiological conditions, both oxygen delivery by RBCs and oxygen consumption by the mitochondria are highly orchestrated processes as part of the normal oxygen homeostasis. This delicate balance is critical since either insufficient or excess oxygen leads to increased levels of damaging reactive oxygen species (ROS) (Zhang et al., 2022). The transcriptional factor HIF-1α (hypoxia inducible factor-1α) plays a critical role in maintaining the oxygen homeostasis in humans by directly controlling the expression of hundreds of target genes. Under normal oxygen tension (normoxia), the transcriptional activity of HIF-1α is halted by hydroxylation catalyzed by prolyl hydroxylase (PHD), a non-heme iron, and α-ketoglutarate-dependent dioxygenase, leading to its ubiquitination, and, finally, degradation by the proteasomal machinery (Semenza, 2007). HIF contains two subunits: an α-subunit and a more stable β-subunit. During the initial stages of hypoxia, the α-subunit quickly degrades in the presence of oxygen (half-life is less than 5 min in 21% oxygen) (Chowdhury et al., 2009).

Almost 95% of all the oxygen consumed by tissues is driven by mitochondrial respiration (Pittman, 2011). A recent example of how the disruption in the blood oxygen content impacts oxygen hemostasis was demonstrated in a COVID-19 hamster infection model. In this model, we reported that HIF-1α’s transcriptional pathways were activated, perhaps due to a lack of oxygen or an accumulation of mitochondrial reactive oxygen species in the lungs of adult Syrian hamsters infected with SARS-CoV-2 (Jana et al., 2022).

In this report, we measured changes in oxygen-binding parameters during the 42-day storage of RBCs and correlated them with the changes in tissue oxygen-sensing pathways and the mitochondrial bioenergetics in human pulmonary arterial endothelial cells (HPAECs). The oxygen dissociation curves (ODCs) demonstrated the expected increase in the oxygen affinity with time, whereas the Hill number (*n*) was unchanged during storage. There was a 30% reduction in P_50_ (oxygen saturation when Hb is half-saturated) and a marginal change in the Bohr effects (pH effects) in the older versus younger ones. The P_50_ values that lowered due to age corresponded to a progressive increase in the expression of HIF-1α and mitochondrial oxygen consumption rates when RBCs at different ages were incubated with pulmonary endothelial cells. There was a considerable increase in the spontaneous oxidation of intraerythrocytic Hb at 37°C, but under oxidative stress conditions, there was a clear alteration in band 3 proteins, which can be attributed to the formation of higher oxidation intermediate, ferrylHb.

## Materials and methods

### Materials

All chemicals and reagents were purchased from Sigma-Aldrich (Saint Louis, Missouri) or Fisher Scientific (Pittsburgh, Pennsylvania), unless otherwise specified. Hydrogen peroxide (H_2_O_2_) (30% w/w) was purchased from Sigma-Aldrich. Fresh solutions of H_2_O_2_ were prepared for every experiment from a stock solution by making appropriate dilutions with deionized water. The solutions were stored on ice. The concentration of H_2_O_2_ was determined spectrophotometrically at 240 nm using a molar extinction coefficient of 43.6 M^−1^ cm^−1^. Buffer solutions were prepared by mixing monobasic and dibasic potassium phosphate dissolved in deionized water, and the pH was adjusted appropriately.

### Blood collection and storage of RBCs

Blood samples used in this study were obtained from healthy donors from the National Institute of Health (NIH) Blood Center, Bethesda, Maryland (FDA/CBER, IRB protocol 03084B) [amendment 03–120B (for red cells)], approved by the Research Involving Human Subjects Committee (RIHSC 2021-CBER-041). For RBC storage, approximately 50 mL of whole-blood was first passed through a neonatal high-efficiency leukocyte reduction filter and then centrifuged at 2000 *g* for 10 min to separate RBCs from the plasma. After the removal of the plasma and the top buffy coat, packed RBCs were stored at 4°C in a 100-mL capacity red blood cell storage bag containing 11 mL of the AS-3 storage solution for up to 42 days, following a standard blood banking protocol.

### Hemoglobin measurements by spectrophotometry

Spectrophotometric measurements were carried out using a UV-visible diode array spectrophotometer (Agilent HP 8453). The levels of ferrous, ferric, and ferrylHbs were measured based on the absorbencies at *λ* = 541, 576, 630, and 700 nm using the published extinction coefficients (Meng and Alayash, 2017). Hb concentrations were calculated based on the heme concentration.

### Hydrogen peroxide-mediated oxidation and ferryl hemoglobin formation

A volume of 1 mL of the stored blood was taken at specific times (1 day, 7 days, and 14 days up to 42 days) for photometric analyses. The solution was then washed with 2 mL PBS, gently stirred, and then centrifuged for 5 min at room temperature. The supernatant was removed; this was repeated twice. RBCs were lysed by adding 3 mL of water, gently stirred, and left to stand for 10 min at room temperature. A measure of 24 mg NaCl was added to the lysate, and the lysate was centrifuged at 4000 *g* for 10 min. The supernatant was filtered with a 0.2-μM filter to remove the RBC membrane. The solution was concentrated, and the Hb concentration was measured.

Spectral changes due to the H_2_O_2_-mediated reaction of Hb (60 μM, heme) were monitored using a photodiode array spectrophotometer using H_2_O_2_ (20 mM), in a measure of 1 mL of solutions at room temperature for 5 min incubation time. FerrylHb formation was followed by monitoring characteristic absorbance changes over time in the visible region (Kassa et al., 2017). In some cases, 1 mL of stored blood was incubated for 1 h at 37 °C with various concentrations of caffeic acid or ascorbate (0.5 µM or 2.5 µM), known for their anti-ferryl activities (Dunne et al., 2006; Kassa et al., 2021). For the verification of the ferryl intermediate, 2 mM sodium sulfide (Na_2_S) was added to transform ferrylHb to sulfhemoglobin (sulfHb). The formation of sulfHb can be monitored by the appearance of an absorbance band at 620 nm and was estimated using the published extinction coefficient (Berzofsky et al., 1971). Spectra were captured at each stage of Hb transformation (i.e., oxyHb, ferrylHb, and sulfHb).

### Measurement of oxygen dissociation curves

ODCs for RBCs were obtained using the Hemox-Analyzer (TCS Scientific, New Hope, PA). The ODC of suspensions of cells was determined, as previously described (Gelderman et al., 2010). To measure ODCs, samples from the stored blood were taken, washed, packed, and resuspended in the plasma to a hematocrit of approximately 20%. Approximately 120 μL of each suspension was added to 3 mL of the Hemox buffer, with a pH of 7.4 in a cuvette and subjected to ODC analysis at 37 °C.

### Endothelial cell culture

Cryopreserved human pulmonary arterial endothelial cells (Thermo Fisher Scientific, Waltham, MA) were cultured in a specially formulated media (Medium 200) containing 2% fetal bovine serum (FBS) supplemented with the Low Serum Growth Supplement (LSGS) (Thermo Fisher Scientific, Waltham, MA). For all experiments, HPAECs were used between passages 5 and 10.

### Effects of hypoxia on the treatment of HPAECs in the presence of RBCs

HPAECs were grown to 80%–90% confluency in complete media before any treatment. Cells were then kept under hypoxia (1% oxygen and 5% CO_2_) conditions for 24 h inside a hypoxia chamber (Plas Labs, Lansing, MI). Cells were exposed to either pre-oxygenated fresh RBC or aged RBC (28-day-, 35-day-, or 42-day-old) for 30 min. All RBC solutions were diluted to 20% hematocrit in cell-culture media and kept on a shaker at 37⁰C for 2 h before incubation with hypoxic HPAECs. A separate RBC-free cell culture medium maintained in a similar oxygenated condition was also added to hypoxic HPAECs to serve as an empty media background. After incubation, cells were taken out the hypoxia chamber and were washed in PBS to remove RBCs. Cells were then immediately lysed with RIPA lysis and extraction buffer (Thermo Fisher Scientific, Waltham, MA) containing the protease inhibitor for further studies.

### Gel electrophoresis, immunoblotting, and fluorescence microscopy

Cell lysate proteins were resolved by SDS-PAGE using precast 4%–20% NuPAGE Bis–Tris Gels (Thermo Fisher Scientific, Waltham, MA) and then transferred to nitrocellulose membranes (Bio-Rad, Hercules, CA) using the standard immunoblotting technique. Nitrocellulose membranes were processed with different specific primary antibodies, e.g., anti-HIF-1α (ab51608), anti-β actin (ab8227), anti-band 3 (ab108414), anti-phosphotyrosine (ab179530), and anti-phospho (Y359)–band 3 (ab77236) (Abcam, Cambridge, MA, USA). Appropriate HRP-conjugated goat anti-mouse IgG (ab97040) and anti-rabbit IgG (ab205718) secondary antibodies were also obtained from Abcam (Cambridge, MA).

### Microparticle preparation from aged RBCs

According to the current US Food and Drug Administration (FDA) regulations, we stored packed RBCs (pRBCs) up to 42 days. At the end of each storage period, e.g., 28 d, 35 d, and 42 d, MPs were isolated from the aged pRBC units through serial centrifugation, following a procedure published by Kim et al. (2020). Since fresh RBCs do not produce enough MPs, we only used 28 d and older pRBCs for MP preparation. For MP toxicity studies on HPAECs, pelleted MPs were resuspended in the endothelial cell culture medium and stored at −80°C. For comparison purposes, MPs were expressed as total MPs obtained from each ml of pRBCs.

### Measurement of ATP and protein carbonylation in aged RBCs

Intracellular ATP levels in fresh and stored RBCs were measured using a luminometric ATP assay kit from Abcam (Cambridge, MA), following a method previously published (Cloos et al., 2020). The protein carbonyl content in RBC lysates was assessed using a dinitrophenylhydrazine (DNPH)-based assay kit (ab126287) as a measure of protein oxidation (Abcam, Cambridge, MA, USA). In the protein carbonyl assay, carbonyl groups in protein side chains are derivatized to DNP hydrazone, following the reaction with DNPH. The absorbance of DNP hydrazones formed in this reaction was measured at 375 nm using a BioTek Synergy HTX microplate reader (Agilent, Santa Clara, CA).

### Endothelial bioenergetic and glycolytic flux measurements

The cellular oxygen consumption and glycolytic rate in HPAECs were assessed in real time using an Agilent Seahorse XF24 Extracellular Flux Analyzer (Agilent, Santa Clara, CA), as previously described. Briefly, 80,000 cells/well were cultured in collagen I-coated 24-well XFV7 cell culture plates (Agilent, Santa Clara, CA) for 24 h. Each well was treated with MPs obtained from 1 mL of RBCs (28-d-, 35-d-, or 42-d-old). Following the incubation with MPs from various aged RBC groups, media were gently washed once with PBS and replaced with 500 µL of XF assay media (Agilent, Santa Clara, CA) supplemented with 10 mM glucose, 5 mM pyruvate, and 2 mM glutamate. The mitochondrial oxygen consumption rate (OCR) was assessed under different bioenergetic states, e.g., coupled, uncoupled, and inhibited states created by automated sequential injections of oligomycin (1 µM), carbonyl cyanide-p-trifluoromethoxyphenylhydrazone (FCCP, 1 µM), and a combination of mitochondrial inhibitors (rotenone, 1 µM and antimycin A, 1 µM), respectively.

Similarly, the endothelial glycolytic capacity was assessed by measuring the extracellular acidification rate (ECAR). For ECAR experiments, glucose-free XF assay media were used. The real-time glycolytic profile was obtained by the sequential addition of glucose (10 mM), oligomycin (1 µM), and glycolytic inhibitor 2-deoxyglucose (2-DG, 100 mM) to the wells. The OCR and ECAR values were plotted using XF24 software, version 1.8. To eliminate any background OCR or ECAR, few blank wells without any cells were also run. Various bioenergetic and glycolytic parameters were calculated, following the manufacturer’s protocol and as described previously. Briefly, basal respiration was considered the difference between the maximum OCR obtained before oligomycin addition and the non-mitochondrial OCR obtained after rotenone/antimycin A, whereas maximal respiration was the difference between the maximum OCR induced by FCCP and the non-mitochondrial OCR. Similarly, glycolysis was considered the maximum ECAR obtained after the addition of glucose, and the glycolytic capacity was the maximum ECAR achieved through oligomycin addition that shuts down ATP generation oxidative phosphorylation.

Mitochondrial superoxide generation was measured in HPAECs by MitoSOX Red (Thermo Fisher, USA) using a M200 cell culture media containing pyruvate (2 mM) and succinate (2 mM) following a method published earlier (Jana et al., 2021).

## Proteomic analysis

### Sample preparation

Protein extraction from RBC lysates was carried out using a lysis buffer (8 M urea, 50 mM Tris HCl, pH 8.0, 150 mM NaCL, and 1× Roche cOmplete Protease Inhibitor). Sonication was carried out using a Qsonica sonic probe with the following settings: amplitude 50% and pulse 10 × 1s. The lysate was incubated at room temperature for 1 h with mixing at 1,000 rpm in an Eppendorf thermomixer. The lysate was clarified by centrifugation at 10K rpm for 10 min at 25°C.

### Proteolysis of the extracted protein

A measure of 20 µg of each sample was reduced with 14 mM dithiothreitol at 25°C for 30 min, followed by alkylation with 14 mM iodoacetamide at 25°C in the dark. Proteolysis was carried out using 2.5 µg trypsin (Promega Sequencing Grade) at 37°C overnight. The proteolyzed samples were cooled to room temperature. The volume of the sample was brought to 0.5 mL with ammonium bicarbonate. The proteolyzed samples were centrifuged at 10,000 × g and desalted using a Waters HPB solid phase extraction plate. The samples were lyophilized and reconstituted with 0.1% TFA prior to MS analyses.

### Mass spectrometry

Mass spectrometry experiments were carried out at BioWorks Laboratories (Ann Arbor, MI). The equivalent of 1 µg of each digest was analyzed by nano LC-MS/MS with a Waters nanoACQUITY UPLC system interfaced with a Thermo Fisher Fusion Lumos Mass Spectrometer. Peptides were loaded on a trapping column and eluted over a 75-µm analytical column at 350 nL/min with a 2-h reverse phase gradient; both columns were packed with Luna C18 resin (Phenomenex). The mass spectrometer was operated in a data-dependent mode, with Orbitrap operating at 60,000 FWHM and 15,000 FWHM for MS and MS/MS, respectively. The instrument was run with a 3-s cycle for MS and MS/MS. Here, advanced precursor determination (APD) was employed.

### Proteomic data analysis

Raw files from the mass spectrometric analysis were converted to the mgf file format, prior to searching against the Swiss-Prot database for protein identification. The database search was carried out with the following parameters: two missed cleavages, peptide tolerance 10 ppm, MS/MS tol. ± 0.1 Dalton, variable modification (C) carbamidomethylation, (M) oxidation, (M) deoxidation, (C) trioxidation, (H W) oxidation, and peptide charge = 1^+^, 2^+^, and 3^+^. The data files from Mascot were then submitted to Scaffold for peptide and protein validation using “Peptide Prophet” and “Protein Prophet”. Probabilities were set to 95% for peptide identification and 90% for protein identification. Label-free identification was carried out using Scaffold’s “weighted spectral counting method.” The volcano plot was generated by Scaffold, and the heatmap was generated using Scaffold LFQ.

### Statistical analysis

Plotting of raw data and all statistical calculations were carried out using GraphPad Prism 8 software. All values are expressed as mean ± SD and error bars in bar diagrams, which are indicative of SD. A *p*-value of <0.05 was considered statistically significant. The difference between two means was compared using paired Student’s *t*-test.

## Results

### Storage of RBCs reduces the oxygen binding affinity of intraerythrocytic hemoglobin

**FIGURE 1:**
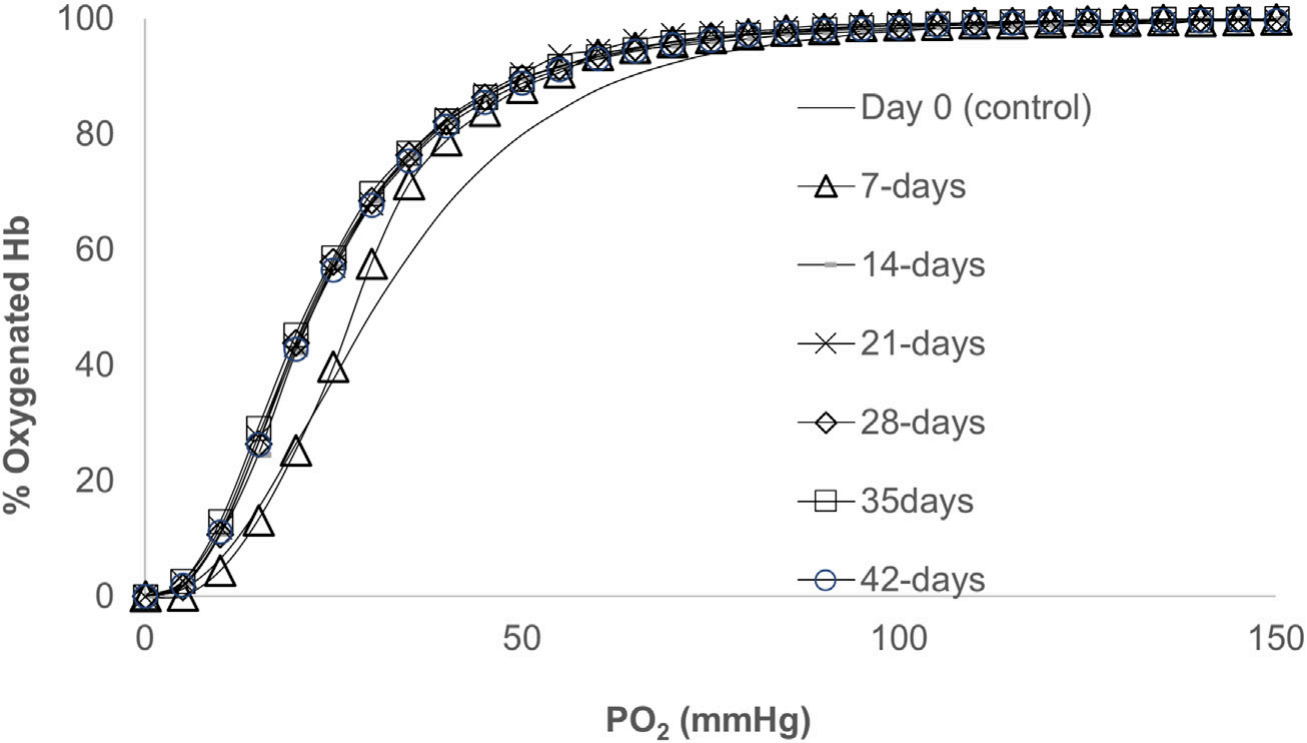
Oxygen dissociation curves (ODCs) for blood stored over a period of 42 days. The ODCs were measured using a Hemox-Analyzer (TCS Scientific). Experiments were carried out with 25% of hematocrit in 3 mL of Hemox solution (135 mm NaCl, 30 mm TES [N-tris (hydroxymethyl)methyl-2-aminoethanesulfonic acid], and 5 mm KCl} (TCS Scientific)) and the anti-foaming agent at 37°C.

Figure 1 shows ODCs obtained from blood samples collected as a function of storage time. As time progressed, ODCs were left shifted but maintained their sigmoidal shape with little change in their cooperativity in keeping with the early reports (Bunn et al., 1969; Gelderman et al., 2010). During the first week of storage in AS-3, a progressive decrease in the oxygen affinity was observed with the largest drop in P_50_, approximately 5 mmHg. However, during subsequent weeks, the P_50_ value remained approximately 20 mmHg throughout the incubation period. A Hill plot of oxygen binding (*n*) was generally linear for values of *Y* between 0.1 and 0.9 (10%–90% saturation). The Hill number (*n*) was unchanged during storage (Figure 1; Table 1), and it remained at >2 throughout the storage period, which indicates that co-operative binding of Hb to oxygen was largely maintained.

**TABLE 1:** Quantitative data representing oxidative reactions and the hemolysis of RBCs together with their hemoglobin oxygen affinities with age.

	Day 0 (control)	Day 7	Day 14	Day 21	Day 28	Day 35	Day 42
Hemolysis%	0.096	0.39	0.77	0.92	0.96	1.22	1.68
OxyHb%	100	100	99.1	98.9	99.1	99.4	99
MetHb% (20°C)	0	0	0.9	1.1	0.9	0.6	1
MetHb% (37°C)	31.1	32.3	32.2	31.1	32.6	35.1	35.7
P_50_ (mmHg)	30.01	25.56	22.12	21.91	22.17	22.08	22.1
Hill coefficient (n_50_)	2.72	2.65	2.5	2.53	2.58	2.5	2.54

We previously reported the oxygen dissociation kinetics from RBCs and CO binding to deoxyRBCs at different age groups using a microvolume stopped-flow instrument and showed little or no change in the kinetic parameters (oxygen binding (*k*_on_) and release (*k*_off_) rates) (Gelderman et al., 2010) in agreement with our current equilibrium data.

### Left-shifted oxygen dissociation curves of aged RBCs correlate with the expressions of hypoxia inducible factor in HPAECs

**FIGURE 2:**
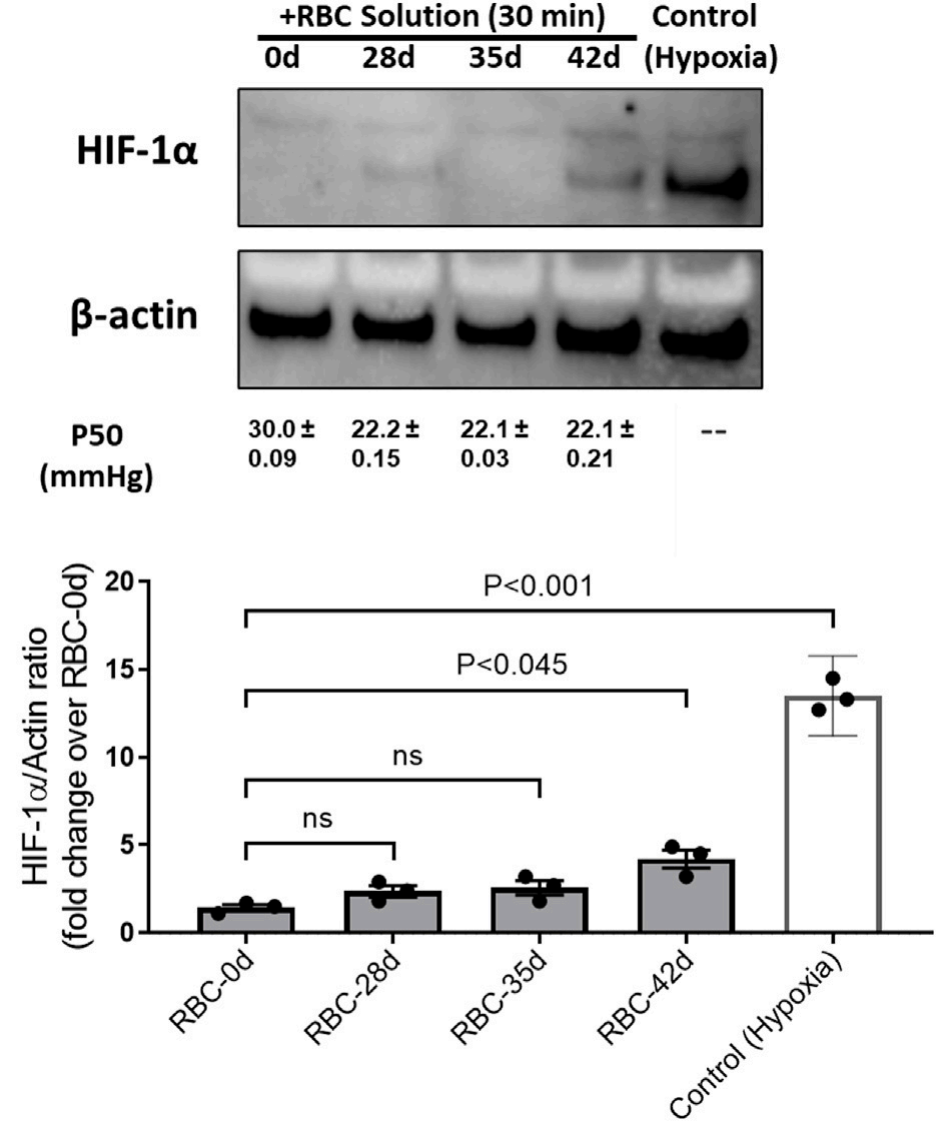
Expressions of the hypoxia-inducible factor (HIF-1α) in human pulmonary endothelial cells under hypoxia and its reversal by fully oxygenated younger red blood cells. HIF-1α expression was induced in HPAECs by subjecting the cells under the hypoxic environment (1% O_2_). Pre-oxygenated RBC solutions of different ages (0, 28, 35, and 42 days old) were added and incubated for 30 min within the hypoxia chamber. Control (hypoxic) cells were lysed immediately within the chamber after hypoxia. Cells were lysed with the RIPA buffer, and cell lysates were analyzed by immune blotting using the anti-HIF-1α antibody. Histogram showing the relative expression of HIF-1α, following normalization with the β-actin protein as the loading control.

We previously demonstrated that a low-oxygen affinity cell-free Hb with a P_50_ value of approximately 40 mmHg suppressed tissue-specific HIF-1α for at least 4–8 h in rats that underwent 80% exchange transfusion of their blood with this oxygen therapeutic mechanism (Manalo et al., 2008). This study was first carried out to demonstrate that in a living organism, a circulating oxygen carrier crosstalk with tissue oxygen-sensing mechanisms by suppressing HIF-1α expressions for a few hours, where oxygen was available before erythropoietin (EPO) rebound, occurred due to the clearance of Hb by the kidneys. In the current experiment, we subjected HPAECs to hypoxia (for 24 h at 1% oxygen) and then exposed HPAECs to oxygenated RBCs of different age groups for 30 min and measured HIF expressions in these cells. As shown in Figure 2, under controlled hypoxic conditions, there was a complete suppression of HIF-1α in endothelial cells incubated with either fresh (0-day) or 28- and 35-day-old RBCs, indicating sufficient and comparable oxygen delivery capabilities in RBCs aged up to 35 days or fresh RBCs. However, when 42-day-old RBCs were incubated with HPAECs, there was a partial suppression in the HIF-1α expression even when the P_50_ values of these old RBCs are equivalent to those of the younger RBCs (P_50_ ∼20 mmHg).

### Spontaneous oxidation (autoxidation) and peroxide-mediated oxidation reactions increase in RBCs over a 42-day incubation period

**FIGURE 3:**
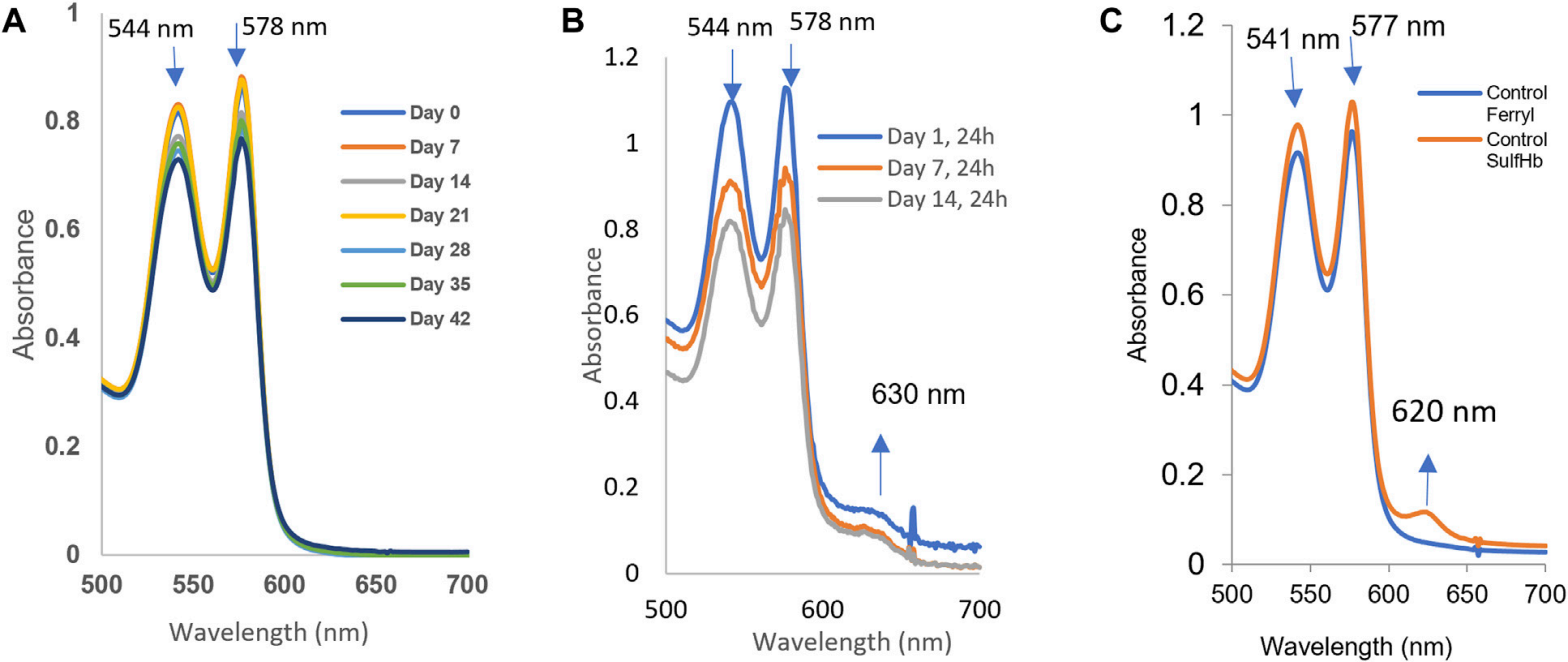
Spontaneous oxidation (autoxidation) and peroxide-mediated oxidation of hemoglobin in fresh and older RBCs. The samples (*n* = 3) from stored blood at different time points (0, 7, 14, 21, 28, 35, and 42 days) were collected and immediately hemolyzed. A measure of 60 µM of hemoglobin samples was prepared, and autoxidation was measured spectrophotometrically. **(A)** Autoxidation experiments were carried out at room temperature, while three of them **(B)** show autoxidation spectral data collected at 37°C from 0 to 14 days. **(C)** Hydrogen peroxide-induced oxidation of a 42-day sample of RBCs. The sample was hemolyzed, and 60 µM of hemoglobin was treated with 20 mM hydrogen peroxide, followed by addition of 2 mM Na2S to derivatize the ferryl formed in the reaction.

The oxidation (in air) of the heme iron of intraerythrocytic Hb (autoxidation) is known to occur with time. We followed Hb’s oxidation in the blood stored under blood bank conditions (4°C) over a period of 42 days. Spectral analyses of samples taken after equilibration at either room temperature, 20°C or 37°C, conditions were carried out at specific time intervals, 0, 7, 14, 28, 35, and 42 days at 20°C (Figure 3A). Table 1 lists the proportions of the oxy and ferric (met) Hb component during the storage time. Under room temperature conditions, these samples generally preserved the oxygenated nature of its protein (Hb) up to approximately 1% over the 42-d time period. However, at the physiological temperature (37°C), there was a considerable accumulation of oxidized Hb, as can be seen in Figure 3B (see table 1), during storage.

We monitored hemolysis with time and found it within the expected range of 1%–2%. It was argued, however, that RBCs under some pathological/storage conditions are exposed to oxidants, such as peroxide in blood (Forman et al., 2016; Alayash, 2022), which can fuel oxidation reactions and oxidative changes in Hb. Figure 3C shows spectral data collected during the exposure of 42-day-old RBCs to 20 mM of hydrogen peroxide. During peroxide-mediated oxidation, a higher oxidation Hb intermediate in the ferryl state (HbFe^4+^) is formed. The ferryl heme is a highly reactive and damaging species of Hb; it is transient in nature as it immediately reverts to the ferric form. To capture ferrylHb, we treated RBC solutions with sodium sulfide (Na_2_S) to derivatize it into more stable sulfHb. We were able to calculate the levels of ferryl in these RBC solutions to be 4.7 µM (heme); Figure 3C.

We have previously documented that ferrylHb, once formed, can target several protein structures within RBC membranes, including band 3 proteins, causing oxidative and post-translational modifications, clustering of band 3, and MP formation (Jana et al., 2018; Strader et al., 2020).

**FIGURE 4:**
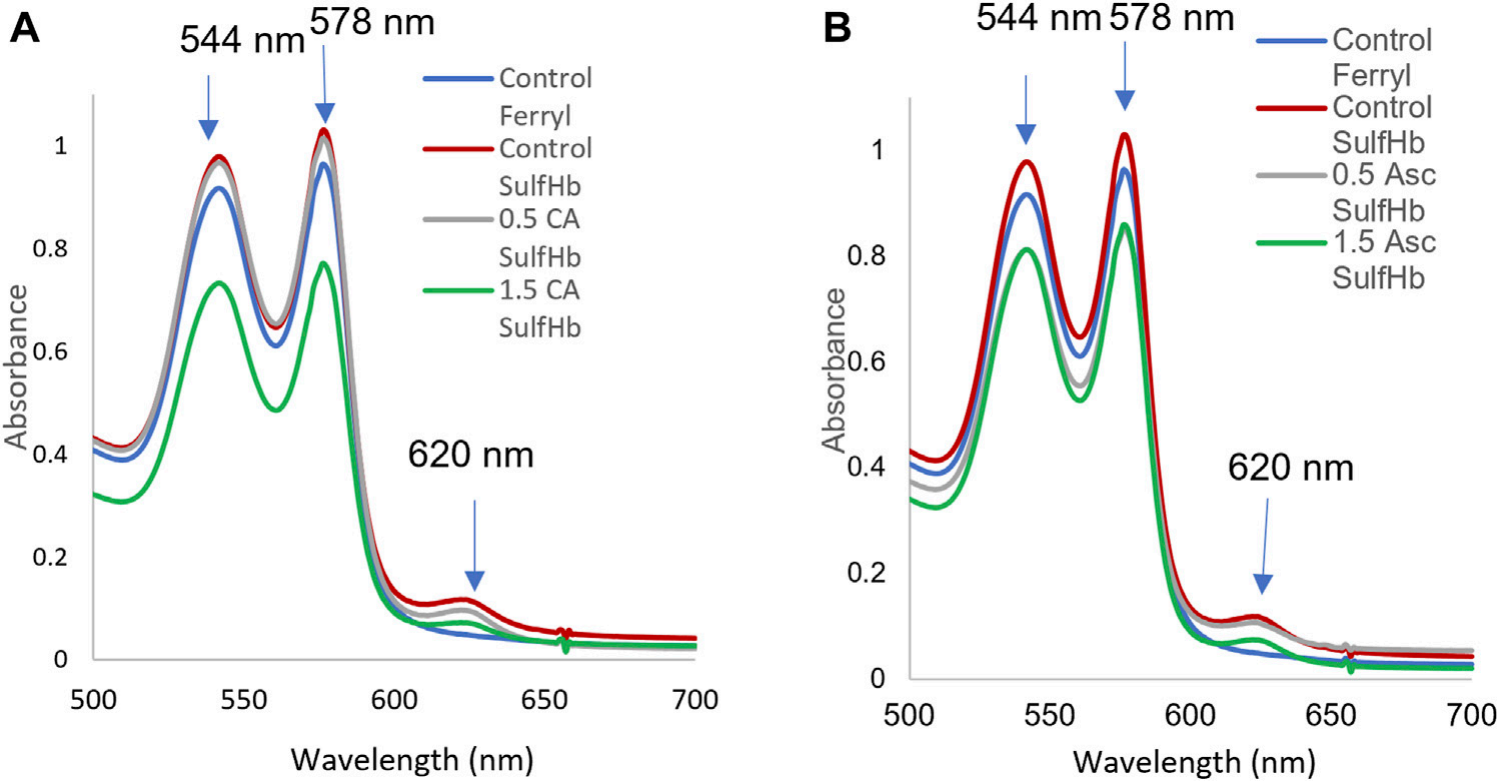
Hydrogen peroxide-mediated oxidation of red blood cells and ferryl hemoglobin reduction by ascorbic and caffeic acids. A stock solution of 60 µM of Hb (per heme) was prepared from the stored blood hemolysates (42 days). The hemoglobin solution was treated with 20 mM H_2_O_2_ for 5 min, followed by an immediate addition of 2 mM Na_2_S to capture the transient ferrylHb species. Spectra were captured at each stage of hemoglobin transformation (oxyHb, ferrylHb, and sulfHb). The ferrous, ferric, and ferrylHbs levels were measured based on the absorbencies at λ = 541, 576, 630, and 700 nm using the published extinction coefficients (Meng and Alayash, 2017). Hemoglobin concentrations were calculated on the heme basis. Control ferryl (blue), control sulfHb (green), 0.5 mM caffeic acid or 0.5 ascorbic acid (gray), and 1.5 mM caffeic acid **(A)** or ascorbic acid (green) **(B)**. Downward arrows indicate changes in the Soret region due to the oxidation of ferrous hemoglobin and ferrylHb formation. Upward arrows indicate the formation of sulfhemoglobin, which absorbs strongly at 620 nm with a subsequent reduction by ascorbic and caffeic acids.

The additional verification of the redox transition of intraerythrocytic Hb oxidation and ferryl formation in aged blood after peroxide treatment was obtained by incubating the 42-day-old RBCs with two antioxidants, ascorbic acid and caffeic acid. These antioxidants are known to interact with RBC membranes and reduce ferryl heme (Kuck et al., 2018; Alayash, 2022). We see a slight reduction in the ferrylHb content, 20% and 28%, by these two acids, respectively. This moderate reduction in the ferryl content may be due to the loss of some of the ferrylHb amount during the spectrophotometric analysis and/or the reduced impenetrability of the RBC membrane to these two reagents (Figure 4, Panel A and B; Table 2).

**TABLE 2:** Reduction of ferryl hemoglobin within red blood cells by ascorbic acid (Asc) and caffeic acid (CA).

	[SulfHb] uM	metHb%
Control	4.76	1.16
0.5 CA	4.46	0.4
1.5 CA	3.43	2.9
0.5 Asc	4.17	0.43
1.5 Asc	3.79	3.4

### RBC storage promotes the phosphorylation of band 3

**FIGURE 5:**
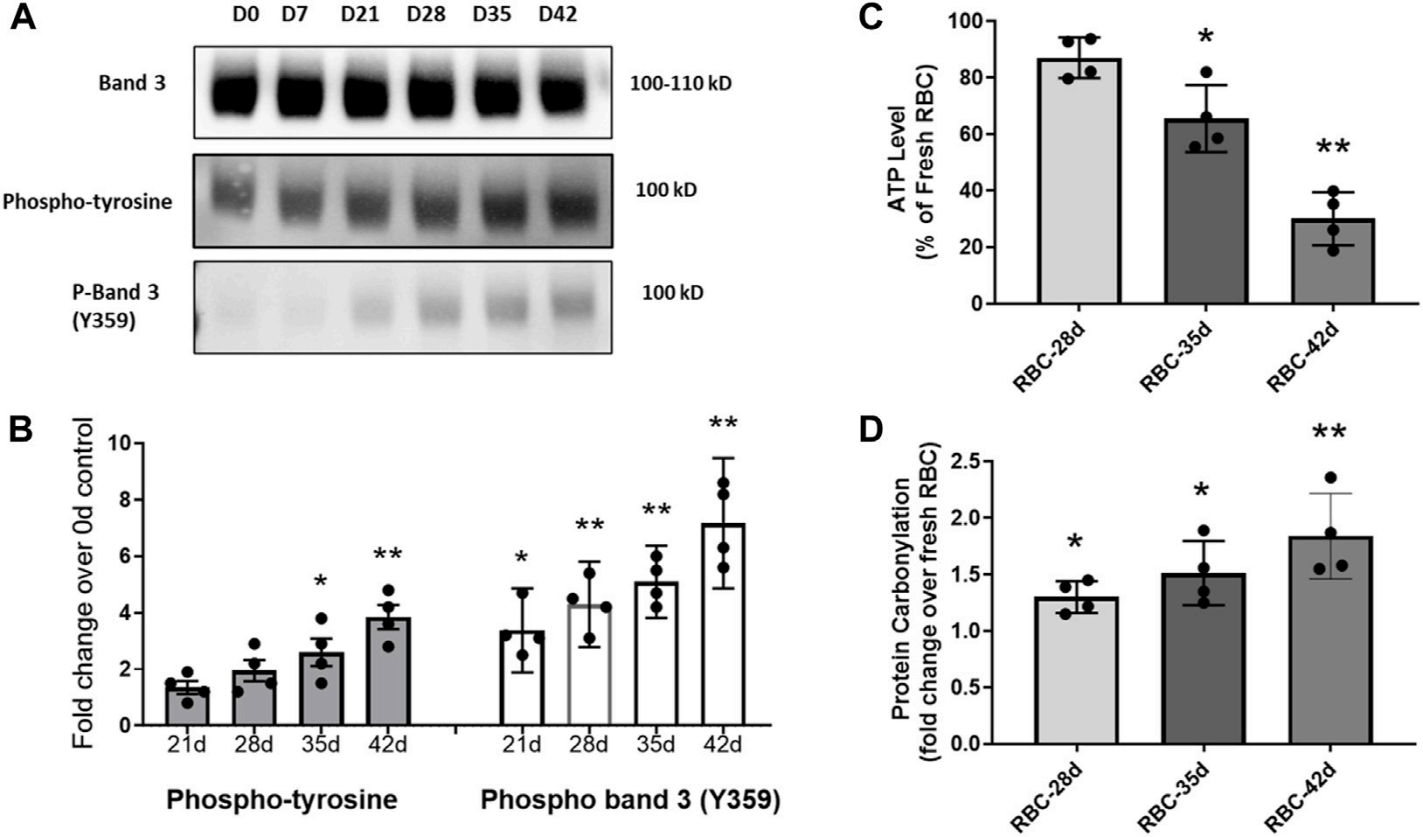
Immunoblot analysis of RBC membrane proteins using an anti-phosphotyrosine antibody and a specific phospho-antibody for band 3 (Y359). **(A)** Immunoblots showing the expression of total band 3, and the levels of total phosphorylated band 3 and phospho-Y359 band 3. **(B)** Histogram shows a progressive increase in the levels of band 3 phosphorylation. **(C)** Histogram showing the basal ATP level in stored RBCs as a percentage of the fresh RBC ATP content. **(D)** Histogram showing a fold increase in protein carbonylation levels in stored RBCs over fresh RBCs. *p <0.05 versus the corresponding fresh RBC values; **p <0.01 versus the corresponding fresh RBC values.

The phosphorylation of RBC’s cytoskeletal and membrane proteins is considered essential for MP formation, and band 3 is one of the most important membrane proteins that undergo extensive phosphorylation under many pathological conditions (i.e., sickle cell disease) and also during RBC storage (Leal et al., 2018). These phosphorylation processes (mostly at tyrosines) act essentially as redox sensors for RBCs (Brunati et al., 2000). We first analyzed RBC membrane proteins for the phosphorylation status of band 3 under different storage durations using standard storage solutions, i.e., AS-3 solutions. Although there was no apparent change in the total band 3 levels, immunoblot analysis using an anti-phosphotyrosine antibody, however, showed a progressive increase in the phosphorylation status of band 3 with the increasing storage time in both RBCs and their derived MPs (Figures 5A, B). There are multiple phosphorylation sites on band 3, and one of the most notable is tyrosine 359. Using a specific antibody for phosphor Y359–band 3, we were able to see a similar trend. We saw an elevation of phosphorylation, mostly after the fifth week, as shown in Figure 5B.

Considerably damaged RBCs at the 42-day-old stage are known to release Hb and microparticles to the medium (hemolysis was 1.9%) at higher rates than young RBCs (Bouchard et al., 2018; Oh et al., 2023). We observed the formation of MPs, mostly after 28 days of storage with a gradual increase in the storage time (data not shown). To assess the metabolic health of aged RBCs, we also measured the ATP levels within RBCs under different storage periods. There was a gradual and steady decline in intracellular ATP with a storage duration dropping to as low as 30% in the 42-day-old RBCs (Figure 5C). The changes in band 3 binding to oxidized Hb depend on Hb oxygenation, Hb autoxidation, glycolysis, and ATP production (De Rosa et al., 2008). We also measured the protein carbonyl formation as an indicator of oxidative stress within stored RBCs, and there was a gradual rise in protein oxidation with the increasing age of RBCs (Figure 5D). These two parameters indicate a steady decline in the RBC cellular metabolic health with a gradual loss of the redox balance (Figures 5C, D).

### Stored RBC-derived MPs impair mitochondrial bioenergetics in human pulmonary endothelial cells

**FIGURE 6:**
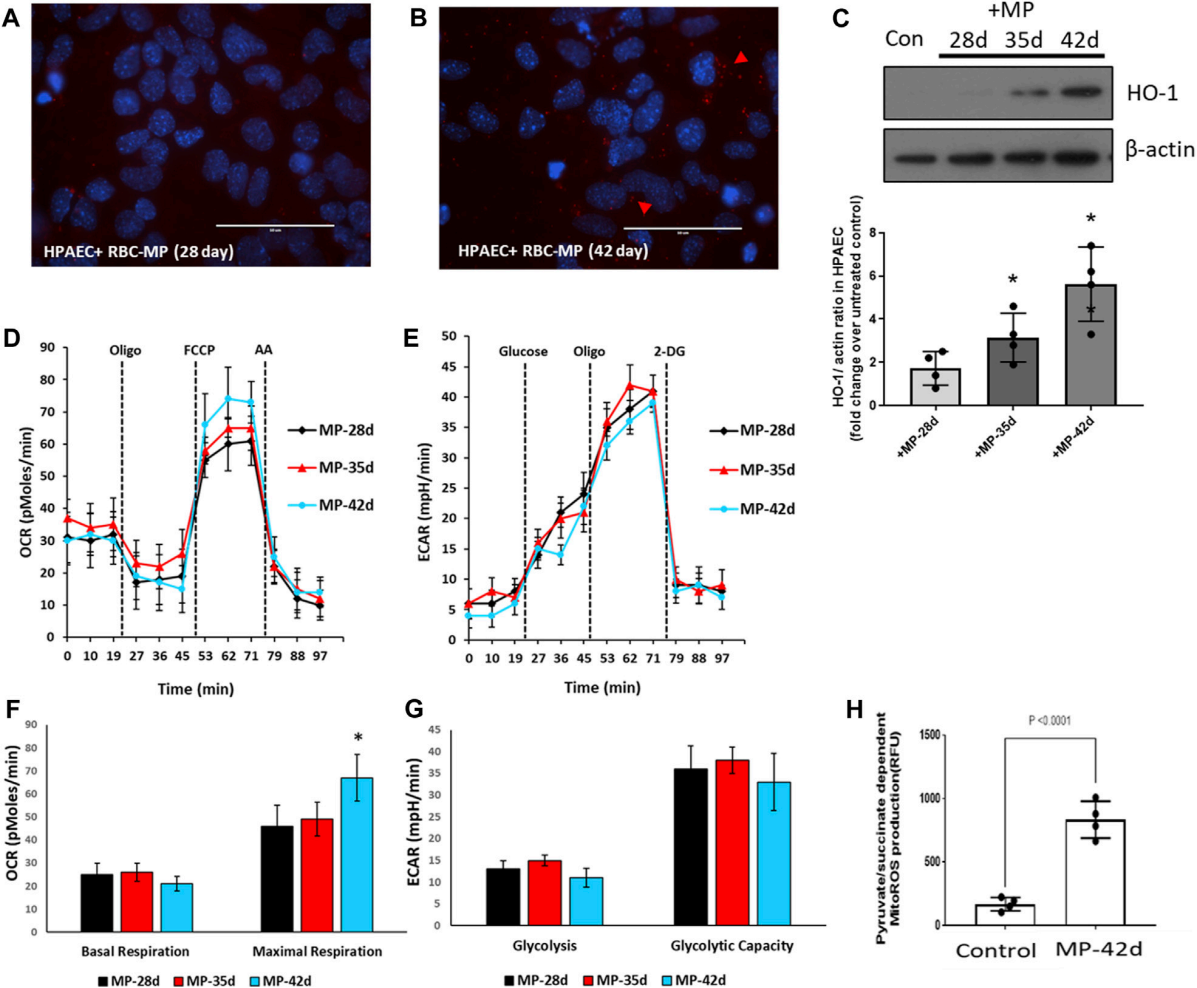
Impact of stored red blood cell-derived microparticles on human pulmonary arterial endothelial cells. MPs were prepared from stored RBCs (28, 35, and 42 days). HPAECs were incubated with MPs obtained from RBCs. **(A,B)** Fluorescent micrographs showing the infiltration of MPs within HPAECs at 28 d and 42 d. **(C)** HO-1 expression, within HPAECs, was monitored by immunoblotting using the anti-HO-1 antibody, following the exposure to stored RBC-derived MPs. Histogram showing the densitometric analysis of the HO-1/actin ratio as the fold increase over untreated control. **(D,E)** Oxygen consumption rates (OCRs) and extracellular acidification rates (ECARs) were obtained from HPAECs by XF analysis to measure the aerobic and anaerobic energy metabolism, following exposure to MPs. **(F,G)** Histograms showing major bioenergetic parameters obtained from OCRs and ECARs. **(H)** Substrate-dependent (pyruvate) mitochondrial ROS production was measured in HPAECs, following exposure to MPs from stored RBCs. *p <0.05 versus the corresponding control.

Due to the complexity of co-culturing RBCs with endothelial cells, we used MPs obtained from aged RBCs and co-incubated them with cultured HPAECs to monitor the effects of stored RBCs indirectly. We separated the MP-rich supernatant from 42-day-old stored RBCs and added them to the HPAEC culture to observe any internalization of the MP content and RBC-derived Hb/heme. The upper fluorescence images in Figures 6A, B show the appearance of RBC-derived MPs in endothelial cells, as indicated by red fluorescent signals from the Alexa Fluor Red tagged band 3 proteins in RBC-derived MPs. We also evaluated heme oxygenase-1 expression (HO-1) (heme scavenger) in these endothelial cells treated with MPs isolated from RBCs of different ages. MPs from 28-d-old RBCs did not induce any significant HO-1 expression, but a progressive increase in HO-1 induction by MPs was seen with an increase in the RBC age, indicating a more oxidized Hb and heme release within the HPAECs by those MPs (Figure 6C).

We next monitored the impact of MPs on pulmonary endothelial bioenergetics. We measured both the aerobic/mitochondrial and anaerobic/glycolytic activities in human endothelial cells exposed to MPs collected from differently aged RBCs. Figures 6D, E show bioenergetic plots obtained using an Agilent Seahorse XF analyzer, which depicts mitochondrial oxygen consumption rates measured in real time in human pulmonary arterial endothelial cells incubated with or without RBC-derived MPs from different storage durations. Bioenergetic parameters calculated from these plots were largely unchanged, except for a rise in maximal respiration, i.e., respiration under the uncoupled state using a protonophore FCCP (Figures 6 F, G). We have previously observed that oxidized Hb and some oxidatively unstable mutant Hbs, e.g., sickle Hb, are capable of inducing a rise in the maximal respiration by possibly releasing heme, which can act as an uncoupler (Chintagari et al., 2016; Jana et al., 2020).

Heme released from oxidatively unstable Hb can cause the activation of TLR4, inducing HO-1, and can, thus, generate carbon monoxide (CO) as a byproduct of heme degradation. Activated TLR4 and CO are both known to cause the uncoupling of mitochondrial respiration (Kaczara et al., 2015; Liu et al., 2015; Kaczara et al., 2016). This mild uncoupling effect can be seen clearly with the 42-day RBC-derived MPs. Another way of identifying mitochondrial impairment is through the measurement of MitoROS. The histogram in Figure 6H shows a considerable rise in the mitochondrial ROS production, indicative of respiratory chain inhibition by RBC-derived MPs. Surprisingly, we found a significant rise in pyruvate (substrate)-dependent mitochondrial superoxide production in cells treated with stored RBC MPs. Since endothelial cells heavily rely on glycolysis as their primary energy source, we assessed the glycolytic activity in those cells by measuring the rates for extracellular acidification. This is an indirect measure of glycolytic lactate production. There was little or no change in ECARs with any RBC-derived MPs, as shown in Figures 6E, G.

### Redox reactions of RBCs modulate the proteome of stored blood

**FIGURE 7:**
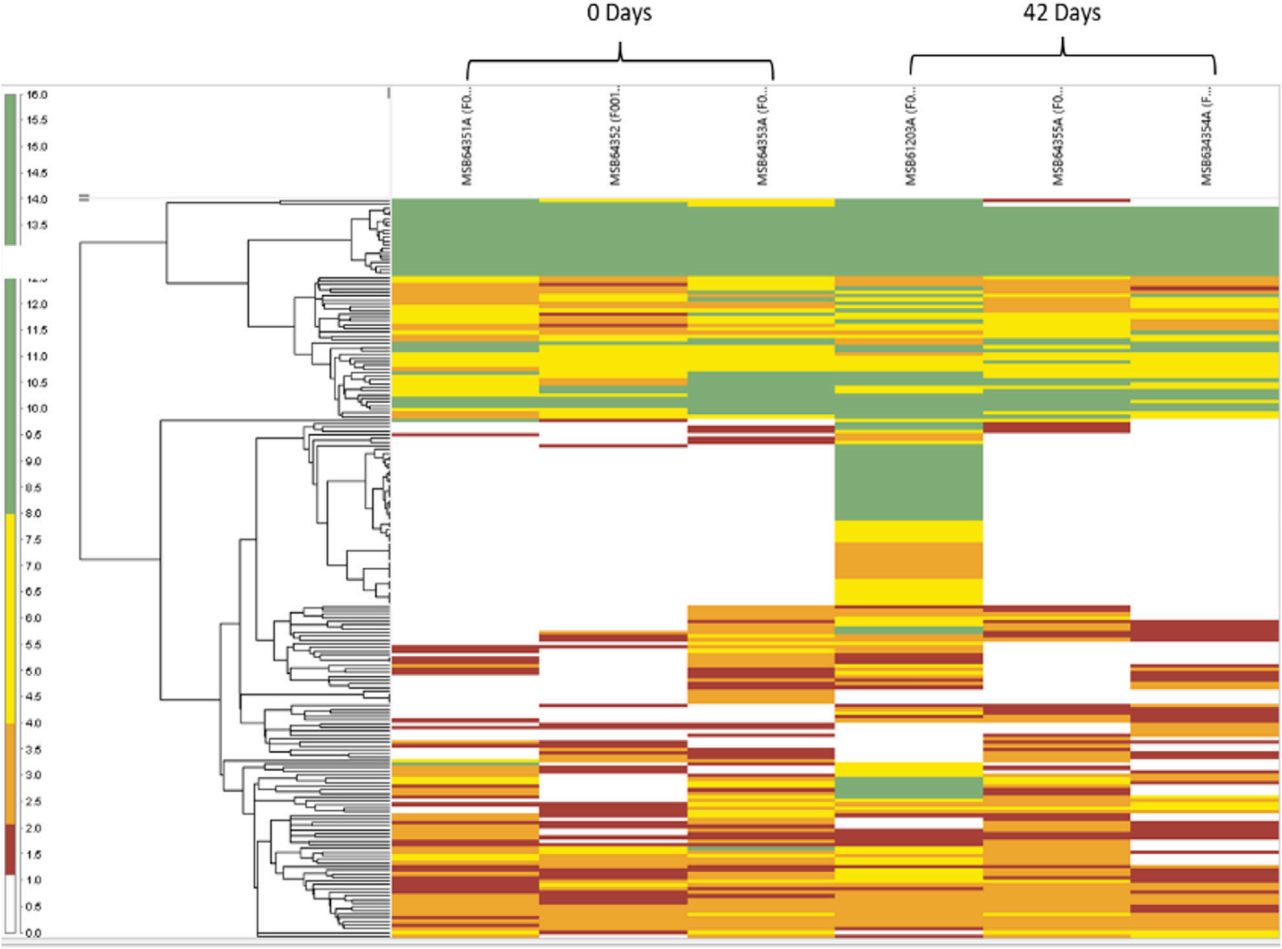
Heatmap reflects the abundance and variance of individual proteins for both the 0- and 42-day time points and within the individual replicates. The branching pattern is seen to the left of the group’s samples in a hierarchal fashion, according to their similarities with each other.

Next, we investigated the proteomic landscape of fresh blood versus blood that was aged for up to 42 days with a focus on those upregulated and downregulated proteins that are specifically involved in the redox metabolism of RBCs. Figure 7 shows heatmap-related changes in the gene expression as a function of age in a matrix format, which can simultaneously provide quantitative patterns across proteins in our samples. The colors correlate with the number of spectra associated with an individual protein expressed as weighted spectral counts, where green represents a higher abundance (8.0–16.0 spectra) and red represents the lowest abundance (1.0–2.0) at two time points, 0 days and 42 days. The two time points are represented by three replicates each.

**FIGURE 8:**
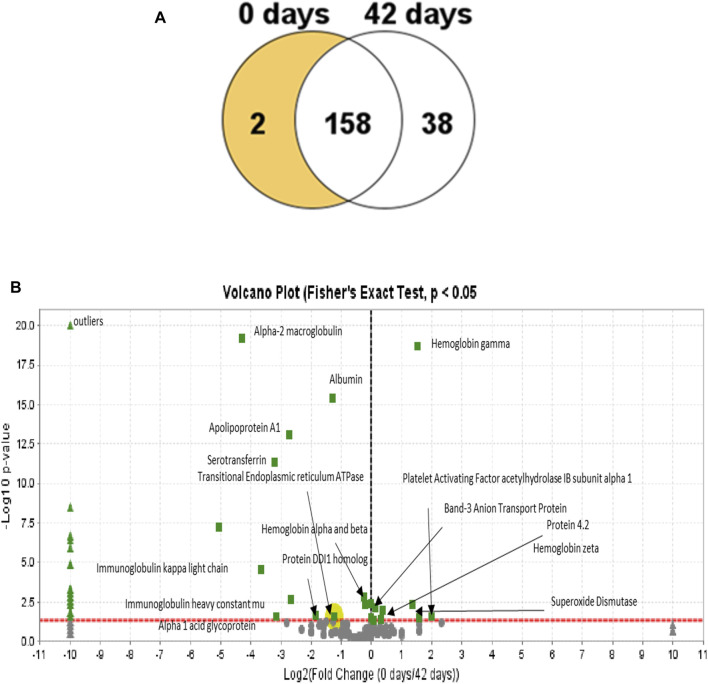
Venn diagram showing the distribution of proteins in the RBC lysates from both time points. **(A)** Diagram shows that most of the proteins are unchanged at 42 days relative to the 0-day time point with only two proteins that are decreased at 42 days and 58 days that were observed to increase. **(B)** Volcano plots (0 and 42 h) represent the average relative fold differences determined by plotting *p* values (–log_10_) for each protein against the calculated fold change (log_2_) difference. The *X*-axis shows the log of the fold change between the two timepoints. The *Y*-axis is the negative log of the *p*-value. Points above the red line represent significant changes. Points to the left of the *Y*-axis are enriched after 42 days, and points to the right are decreased at 42 days.

In Figure 8A, the Venn diagram shows the difference in the number of proteins grouped according to the RBC’s age. A total of 198 proteins were found across the samples, with 38 proteins exhibiting a statistically significant abundance change (*p*-value <0.05) at 42 days and only two proteins at 0 days. Our samples were leukoreduced and include two different time points. Fewer proteins were increased with the storage time, compared with the RBC supernatant. The increased proteins include proteasome subunits, glycolytic enzymes, and redox and other intracellular proteins. This is consistent with the previous reports (Dzieciatkowska et al., 2013; Bryk and Wiśniewski, 2017). Many proteins identified in this study were common plasma proteins, including albumin, α- and β-Hb chains, and enzymes, involved in the essential metabolic function, such as glycolysis.

Proteomic analysis of the 0- and 42-day time point mass spectrometric data was carried out using Scaffold software. A Scaffold volcano plot (Figure 8B) provides a graphic representation of upregulated/downregulated proteins. The volcano plot shows a scatter plot, in which the statistical significance (*p*-value) is plotted versus the magnitude of change (fold change). Among the upregulated proteins, shown in the left panel, were plasma proteins contaminating the samples, even after leukoreduction and the spinning of the plasma. We estimated that at least 8%–10% contaminating proteins in the samples included albumin and Hb.

One of the most intriguing upregulated proteins found is the transitional endoplasmic reticulum ATPase. Transitional endoplasmic reticulum ATPase (also known as p97) is part of the ubiquitin-proteasome system (UPS) essential for rapidly clearing ROS-damaged membrane proteins and maintaining cellular homeostasis in RBCs. A dysfunction in p97 underlies impaired UPS and contributes to oxidative stress in old RBCs. Unlike other nucleated cell types, protein quality control in mature RBCs relies solely on the ubiquitin-dependent proteasome system and not the autophagic–lysosomal pathway (ALP). The detrimental role of dysfunctional p97 was reported to result in the accumulation of polyubiquitinated membrane proteins, oxidative stress, and sickling. Hence, proteasome activator complex subunit 1, like p79, has been described as a repair or “destroy” protein and is recruited to the membrane during storage (Song et al., 2022).

Another upregulated protein is the DDI1 homolog protein, which acts as a proteasomal shuttle and links the proteasome and replication fork proteins, like RTF2. RTF2 is required, with DDI2, for cellular survival, following replication stress. Together or redundantly with DDI2, DDI1 removes RTF2 from stalled forks to allow cell cycle progression after replication stress and maintains the genome integrity (Kottemann et al., 2018).

Among the proteins of interest that were marginally decreased is protein 4.2, an RBC membrane protein. This protein is an ATP-binding protein, which may regulate the association of band 3 with ankyrin. It probably plays a role in the RBC shape and mechanical property regulation (Jay, 1996). Another related protein, the band 3 anion transport protein, has also been downregulated. Band 3 anion transport protein, also known as anion exchanger 1 (AE1) or band 3 or solute carrier family 4 member 1 (SLC4A1), is a protein that is encoded by the *SLC4A1* gene in humans. The band 3 anion transport protein is a phylogenetically preserved transport protein responsible for mediating the exchange of chloride (Cl^−^) with bicarbonate (HCO_3_^−^) across plasma membranes. Another notable reduction is the level of a major antioxidant enzyme, superoxide dismutase (SOD). This enzyme catalyzes the dismutation of the superoxide (O^.−^_2_) radical into molecular oxygen (O_2_) and hydrogen peroxide (H_2_O_2_). In response to an external stimulus, the upregulation of proteins is known to occur as a complementary process that involves an increase in the quantities of cellular proteins (Atkinson and Halfon, 2014). Oxidative stress associated with RBC aging may have triggered the upregulation of some of the proteins unique to the RBCs reported here. However, it is also possible that some low levels of contaminating leukocytes could have contributed to some of the proteins captured in our proteomic analysis (Achilli et al., 2011).

## Discussion

RBC transfusions are life-saving therapy designed to restore or maintain tissue oxygen homeostasis, and therefore, the quality of transfused RBCs is critical to achieve tissue oxygenation. Blood banking storage conditions requires the maintenance of blood at a low temperature in preservation solutions for up to 42 days. During storage, however, RBCs undergo several biochemical and biomechanical changes that reduce RBCs’ survival and compromise the oxygen transporting function of blood. These changes are collectively referred to as the “storage lesion” (D'Alessandro et al., 2010). The transfusion of older RBCs results in the disruption of oxygen homeostasis, primarily due to (a) the clearance of allogenic transfused RBCs using the reticuloendothelial system and (b) the depletion of 2,3-diphosphoglycerate (2,3-DPG) in stored RBCs, altering the ability of Hb to bind and release oxygen during the initial hours posttransfusion (MACDONALD, 1977). Moreover, the unique oxidative milieu prevailing within older RBCs, microparticle formation, and the loss of deformability also contribute to the storage lesion. One of the hallmarks of oxidative changes associated with aging is the oxidation of the spectrin–actin–protein 4.1 and the loss of band 3, an intrinsic RBC membrane protein responsible for the vesiculation of the RBC membrane associated with aging (Bosman et al., 2010).

We have recently shown that Hb oxidation reaction intermediates play a key role in initiating similar RBC membrane changes in sickle cell disease (SCD) RBCs, which share common features with old normal RBCs (Jana et al., 2018; Strader et al., 2020). Specifically, we found that ferrylHb, with a high oxidation state, directly targets band 3 and its associated proteins. The complex formation between the Hb and band 3 network of proteins (which exhibited more phosphorylation and ubiquitination) were shown to be involved in MP formation in the blood from SCD mice and patients (Jana et al., 2018; Strader et al., 2020).

Although the storage lesion has been well-documented for decades (Kim-Shapiro et al., 2011), our understanding of the mechanisms involved in these changes and the clinical consequences remains incomplete. Recent clinical trials and animal experiments have raised fundamental questions about the efficacy of stored RBCs, which may have important implications for the future of transfusion research (Tinmouth et al., 2006; Bertolone et al., 2022).

Our working hypothesis in this manuscript was that blood aging disrupts normal communications between oxygen transport (RBC/Hb), tissue oxygen sensing mechanisms (HIF/PHD), and mitochondrial respiration. Consequently, mitochondrial bioenergetics machinery shifts toward glycolysis with a resultant decrease in intracellular levels of NADPH and ATP. Our investigation was carried out in a simple cell culture system with a limited number of variables, as opposed to animal or human model systems to better understand the interplay between oxygen transport, oxygen sensing, and mitochondrial function, and the impact of RBCs’ age on these pathways.

The oxygen dissociation data obtained from different blood samples stored for 42 days in this report were carried out in AS-3 solutions. These automated measurements carried out using the Hemox-Analyzer are based on the multi-wavelength photometric determination of the oxygen saturation of Hb. This instrument has overcome several limitations associated with older instruments, including the light-scattering effects associated with red blood cell suspensions. The data generally agree with previously reported studies obtained by serial sampling during storage (Bunn et al., 1969; Gelderman et al., 2010). The loading and unloading of oxygen to Hb are described by the sigmodal oxyHb dissociation curve, which enables both efficient oxygen loading at high oxygen tensions in the lungs and the efficient unloading at low partial pressures of oxygen (pO_2_) in microcirculation.

Overall ODCs maintained their sigmoidal (S) shape with the complete saturation of approximately 100 mmHg. However, there was a clear right shift in these curves as the storage time progresses. Cooperativity was also largely maintained with a slight reduction in the calculated (*n*) values. It has been estimated that at least 20%–30% of Hb should be in the “inactive” form to allow for this increase in the oxygen affinity (left shift) (Bunn et al., 1969).

Our data showed that there was little change in metHb levels with the increasing incubation time at room temperature (up to 1%), but a considerably more metHb amount was formed (up to 30%–35%) in RBCs stored at 37°C (Table 1). Equally puzzling is the question of what physiological ramifications that cause a drop in the P_50_ value (∼10–11 mmHg) during the aging process (from the start, *t* = 0 d with P_50_ ∼30 mmHg to the end and *t* = 42 d with P_50_ ∼20 mmHg) will have on oxygen homeostasis in humans?

To understand the consequences of this change, in the oxygen affinity on RBCs (age 0–42 days), we measured HIF-1α in HPAECs exposed to hypoxia (1% O_2_). HIF-1α is a master regulator of oxygen, and its activity is tightly regulated depending on the oxygen concentration in tissues. After 30 min of incubation, we measured the HIF-1α protein in the cells. HIF-1α was degraded very quickly (within 10–15 min). We saw a similar degradation pattern of the HIF protein in HPAECs exposed to RBCs with 28-day- and 35-day-old RBCs, as seen with fresh RBCs; however, the 42-day-old RBCs showed the partial degradation of HIF-1α. Transfusing guinea pigs’ own blood that was stored up to 14 days (equivalent 42 days in humans), and both HIF-1α and its target gene, *EPO*, were reported to increase at the 14-day period. Subsequently, EPO accumulated in the plasma, indicating a decreased O_2_ availability in the kidneys. Conversely, all variables remained at basal levels in the fresh blood group (Baek et al., 2018). Our observations in cells clearly agree with these experimental observations in this animal model.

Next, we investigated another important change that occurs as RBCs age, that is, Hb’s oxidation that affects not only the ability of RBCs to carry oxygen but also these reactions could be damaging to the RBCs and surrounding tissues. Hemolysis is another consequence of Hb oxidation, and the observed upregulation of the HO-1 (scavenger of heme) expression in HPAECs is believed to be in response to Hb/heme released in the medium. Accordingly, it has been suggested that antioxidants should be added to reduce hemolysis during the cold storage of blood (Czubak et al., 2017). Factors affecting the rate of Hb oxidation during red blood cell *ex vivo* storage include compromised antioxidant activity, high concentrations of glucose in the storage media, and the presence of molecular oxygen (Kanias and Acker, 2010). Using ghost cells from normal and SCD blood, we and the others previously showed a direct complex formation between membrane band 3 and ferrylHb (Welbourn et al., 2017; Jana et al., 2018). The amount of ferrylHb bound to normal erythrocyte membranes was found to increase in a recent study with the incubation time after only 2 h of incubation time (Sztiller et al., 2006). The complex formation and other posttranslational modifications of both Hb and band 3 proteins were also reported (Jana et al., 2018; Strader et al., 2020). Our current data confirmed and showed that despite its transient nature, ferrylHb was formed when older RBCs were exposed to hydrogen peroxide. We also used two antioxidants known for their anti-ferryl actions, namely, ascorbic acid and caffeic acids; both reduced the intraerythrocytic ferrylHb concentration by 20% and 30%, respectively.

To examine the impact of internal oxidation reactions on RBC membranes, we assessed Hb-dependent oxidation reactions on band 3 and other related proteins. In these experiments, we used old (42 days) RBCs and microparticles obtained from the MP-rich supernatant.

We first analyzed RBC membrane proteins for the phosphorylation status of band 3 under different storage times under the standard storage solution, i.e., AS-3. Although there was no apparent change in the total band 3 levels, immunoblot analysis using an anti-phosphotyrosine antibody showed a progressive increase in the phosphorylation status of band 3 with the RBC storage time. There are multiple phosphorylation sites on band 3, and one of the most notable of them is tyrosine 359. Using a specific antibody for Y359–band 3, we were able to confirm a similar elevation in phosphorylation, mostly after the fifth week in both RBC and MPs. Numerous studies have previously shown that aged RBCs can produce MPs due to the altered redox balance within the cells and the phosphorylation-driven clustering of band 3 proteins (Rubin et al., 2012; Hoehn et al., 2015). There are multiple oxidation-dependent death-signaling pathways that are reported in stored RBCs, including autophagy and apoptosis (Thiagarajan et al., 2021). The oxidation-dependent activation of caspases, especially caspase 3, can lead to the cleavage of the cytoplasmic end of band 3 (Mandal et al., 2003). The cleavage affects the interactions of band 3 with cytosolic proteins and the linkage to ankyrin and the cytoskeleton (Mandal et al., 2003; Mohanty et al., 2014).

We used the endothelial energy metabolism as an easily readable platform to monitor the impact of RBC-derived MPs in those cells. Generally, fresh RBCs do not produce MPs (Bouchard et al., 2018). We used MPs obtained from 28-d-, 35-d-, and 42-d-old RBCs to study their effects on the endothelial cell metabolism. Our present study is possibly the first to observe bioenergetic changes in human endothelial cells mediated by MPs generated from aged RBCs. There was a positive correlation between the RBC age and the damage potential of the MPs. Our results, showing the HO-1 protein expression in HPAECs by 42-d-old RBC MPs, possibly indicate heme and/or oxidized Hb release within the cytosol of endothelial cells by those MPs. The same mechanism can also explain the mild uncoupling effect seen in endothelial cells exposed to MPs from 42-day-old RBCs, indicating a significant accumulation oxidized Hb and heme in endothelial mitochondria, as reported previously (Chintagari et al., 2016; Jana et al., 2018; Jana et al., 2020).

We also, for the first time, monitored changes in glycolytic rates under the influence of MPs from aged RBCs, although none of the MPs we studied could alter glycolysis; however, the augmentation of uncoupled respiration by MPs was also associated with more mitochondrial superoxide radical production, further supporting a mechanism linked to higher rates of oxidation and a greater heme release by the Hb contained in those aged MPs. CO is one of the major byproducts of heme catabolism by HO-1 and is known to cause the uncoupling of mitochondrial respiration by opening mitochondria-specific ion channels in the endothelium (Kaczara et al., 2015; Kaczara et al., 2016).

Proteomic analysis of RBC lysates from two groups (*t* = 0 and *t* = 42 days) confirmed the presence of protein alterations, including the downregulation of both bands 3 and 4.1. Furthermore, the major antioxidative enzyme superoxide dismutase (scavenger of superoxide ions, which are produced as result of Hb oxidation and from other sources within RBCs) is downregulated. This confirms enhanced oxidation and oxidative changes seen in Hb and other RBC membrane proteins in older RBCs. Recently, using a compact X-band and EPR spectrometer and a cell-permeant molecular probe (CMH) (which is oxidized by O_2_^•−^ to generate a stable nitroxide (CM^•^)), superoxide generation in RBCs was quantified in murine mice strains, whose RBCs are stored well or poorly in the refrigerator (Palha et al., 2022). We also noticed novel changes in the UPS system in response to oxidative stress with the aging of RBCs, such as ubiquitin-dependent protease and the proteasomal shuttling protein (Song et al., 2022).

In summary, our data show that prolonged RBC storage conditions promote changes in oxygen binding and release that are linked to compromises in oxygen sensing and mitochondrial respiration, and that oxidative damage and band 3 alterations are driven by Hb’s oxidative side reactions. Antioxidative interventions targeting intracellular Hb oxidation reactions and membrane changes may lead to the reestablishment of oxygen homeostasis in old RBCs.

## Data Availability

The raw data supporting the conclusion of this article will be made available by the authors, without undue reservation.

## References

[B1] Achilli C. Ciana A. Balduini C. Risso A. Minetti G. (2011). Application of gelatin zymography for evaluating low levels of contaminating neutrophils in red blood cell samples. Anal. Biochem. 409, 296–297. 10.1016/j.ab.2010.10.019 20971053

[B2] Alayash A. I. (2022). Hemoglobin oxidation reactions in stored blood. Antioxidants 11, 747. 10.3390/antiox11040747 35453432 PMC9027219

[B3] Atkinson T. J. Halfon M. S. (2014). Regulation of gene expression in the genomic context. Comput. Struct. Biotechnol. J. 9, e201401001. 10.5936/csbj.201401001 24688749 PMC3962188

[B4] Baek J. H. Yalamanoglu A. Moon S. E. Gao Y. Buehler P. W. (2018). Evaluation of renal oxygen homeostasis in a preclinical animal model to elucidate difference in blood quality after transfusion. Transfusion 58, 1474–1485. 10.1111/trf.14560 29498054

[B5] Bertolone L. Shin H. K. H. Baek J. H. Gao Y. Spitalnik S. L. Buehler P. W. (2022). ZOOMICS: comparative metabolomics of red blood cells from Guinea pigs, humans, and non-human primates during refrigerated storage for up to 42 days. Front. Physiology 13, 845347. 10.3389/fphys.2022.845347 PMC8977988 35388289

[B6] Berzofsky J. A. Peisach J. Blumberg W. E. (1971). Sulfheme proteins. J. Biol. Chem. 246, 3367–3377. 10.1016/s0021-9258(18)62234-3 4324899

[B7] Bosman G. J. Stappers M. Novotný V. M. (2010). Changes in band 3 structure as determinants of erythrocyte integrity during storage and survival after transfusion. Blood Transfus. 8 (3), s48–s52. 10.2450/2010.008S 20606749 PMC2897190

[B8] Bouchard B. A. Orfeo T. Keith H. N. Lavoie E. M. Gissel M. Fung M. (2018). Microparticles formed during storage of red blood cell units support thrombin generation. J. Trauma Acute Care Surg. 84, 598–605. 10.1097/TA.0000000000001759 29251713 PMC5860947

[B9] Brunati A. M. Bordin L. Clari G. James P. Quadroni M. Baritono E. (2000). Sequential phosphorylation of protein band 3 by Syk and Lyn tyrosine kinases in intact human erythrocytes: identification of primary and secondary phosphorylation sites. Blood 96, 1550–1557. 10.1182/blood.v96.4.1550.h8001550_1550_1557 10942405

[B10] Bryk A. H. Wiśniewski J. R. (2017). Quantitative analysis of human red blood cell proteome. J. Proteome Res. 16, 2752–2761. 10.1021/acs.jproteome.7b00025 28689405

[B11] Bunn H. F. May M. H. Kocholaty W. F. Shields C. E. (1969). Hemoglobin function in stored blood. J. Clin. Investigation 48, 311–321. 10.1172/JCI105987 PMC322222 5764013

[B12] Chintagari N. R. Jana S. Alayash A. I. (2016). Oxidized ferric and ferryl forms of hemoglobin trigger mitochondrial dysfunction and injury in alveolar type I cells. Am. J. Respir. Cell. Mol. Biol. 55, 288–298. 10.1165/rcmb.2015-0197OC 26974230 PMC4979363

[B13] Chowdhury R. Mcdonough M. A. Mecinović J. Loenarz C. Flashman E. Hewitson K. S. (2009). Structural basis for binding of hypoxia-inducible factor to the oxygen-sensing prolyl hydroxylases. Structure 17, 981–989. 10.1016/j.str.2009.06.002 19604478

[B14] Cloos A. S. Ghodsi M. Stommen A. Vanderroost J. Dauguet N. Pollet H. (2020). Interplay between plasma membrane lipid alteration, oxidative stress and calcium-based mechanism for extracellular vesicle biogenesis from erythrocytes during blood storage. Front. Physiology 11, 712. 10.3389/fphys.2020.00712 PMC7350142 32719614

[B15] Czubak K. Antosik A. Cichon N. Zbikowska H. M. (2017). Vitamin C and Trolox decrease oxidative stress and hemolysis in cold-stored human red blood cells. Redox Rep. 22, 445–450. 10.1080/13510002.2017.1289314 28277068 PMC6837575

[B16] D'Alessandro A. Liumbruno G. Grazzini G. Zolla L. (2010). Red blood cell storage: the story so far. Blood Transfus. 8, 82–88. 10.2450/2009.0122-09 20383300 PMC2851210

[B17] De Rosa M. C. Alinovi C. C. Galtieri A. Russo A. Giardina B. (2008). Allosteric properties of hemoglobin and the plasma membrane of the erythrocyte: new insights in gas transport and metabolic modulation. IUBMB Life 60, 87–93. 10.1002/iub.15 18379998

[B18] Dunne J. Caron A. Menu P. Alayash A. I. Buehler P. W. Wilson M. T. (2006). Ascorbate removes key precursors to oxidative damage by cell-free haemoglobin *in vitro* and *in vivo* . Biochem. J. 399, 513–524. 10.1042/BJ20060341 16848758 PMC1615907

[B19] Dzieciatkowska M. Silliman C. C. Moore E. E. Kelher M. R. Banerjee A. Land K. J. (2013). Proteomic analysis of the supernatant of red blood cell units: the effects of storage and leucoreduction. Vox Sang. 105, 210–218. 10.1111/vox.12042 23663258 PMC3744597

[B20] Forman H. J. Bernardo A. Davies K. J. A. (2016). What is the concentration of hydrogen peroxide in blood and plasma? Archives Biochem. Biophysics 603, 48–53. 10.1016/j.abb.2016.05.005 27173735

[B21] Gelderman M. P. Yazer M. H. Jia Y. Wood F. Alayash A. I. Vostal J. G. (2010). Serial oxygen equilibrium and kinetic measurements during RBC storage. Transfus. Med. 20, 341–345. 10.1111/j.1365-3148.2010.01016.x 20534030

[B22] Hess J. R. Greenwalt T. G. (2002). Storage of red blood cells: new approaches. Transfus. Med. Rev. 16, 283–295. 10.1053/tmrv.2002.35212 12415514

[B23] Hess J. R. (2014). Measures of stored red blood cell quality. Vox Sang. 107, 1–9. 10.1111/vox.12130 24446817

[B24] Hoehn R. S. Jernigan P. L. Chang A. L. Edwards M. J. Pritts T. A. (2015). Molecular mechanisms of erythrocyte aging. Biol. Chem. 396, 621–631. 10.1515/hsz-2014-0292 25803075 PMC5673117

[B25] Jana S. Heaven M. R. Stauft C. B. Wang T. T. Williams M. C. D'Agnillo F. (2022). HIF-1α-Dependent metabolic reprogramming, oxidative stress, and bioenergetic dysfunction in SARS-CoV-2-infected hamsters. Int. J. Mol. Sci. 24, 558. 10.3390/ijms24010558 36614003 PMC9820273

[B63] Jana S. Heaven M. R. Alayash A. I. (2021). Cell-free hemoglobin does not attenuate the effects of SARS-CoV-2 spike protein S1 subunit in pulmonary endothelial cells. Int. J. Mol. Sci. 22, 9041. 10.3390/ijms22169041 34445747 PMC8396564

[B26] Jana S. Strader M. B. Alayash A. I. (2020). The providence mutation (βK82D) in human hemoglobin substantially reduces βCysteine 93 oxidation and oxidative stress in endothelial cells. Int. J. Mol. Sci. 21, 9453. 10.3390/ijms21249453 33322551 PMC7763657

[B27] Jana S. Strader M. B. Meng F. Hicks W. Kassa T. Tarandovskiy I. (2018). Hemoglobin oxidation-dependent reactions promote interactions with band 3 and oxidative changes in sickle cell-derived microparticles. JCI Insight 3, e120451. 10.1172/jci.insight.120451 30385713 PMC6238743

[B28] Jay D. G. (1996). Role of band 3 in homeostasis and cell shape. Cell. 86, 853–854. 10.1016/s0092-8674(00)80160-9 8808620

[B29] Kaczara P. Motterlini R. Kus K. Zakrzewska A. Abramov A. Y. Chlopicki S. (2016). Carbon monoxide shifts energetic metabolism from glycolysis to oxidative phosphorylation in endothelial cells. FEBS Lett. 590, 3469–3480. 10.1002/1873-3468.12434 27670394

[B30] Kaczara P. Motterlini R. Rosen G. Augustynek B. Bednarczyk P. Szewczyk A. (2015). Carbon monoxide released by CORM-401 uncouples mitochondrial respiration and inhibits glycolysis in endothelial cells: a role for mitoBKCa channels. Biochimica biophysica acta 1847, 1297–1309. 10.1016/j.bbabio.2015.07.004 26185029

[B31] Kanias T. Acker J. P. (2010). Biopreservation of red blood cells-the struggle with hemoglobin oxidation. Febs J. 277, 343–356. 10.1111/j.1742-4658.2009.07472.x 19968714

[B32] Kassa T. Brad Strader M. Nakagawa A. Zapol W. M. Alayash A. I. (2017). Targeting βCys93 in hemoglobin S with an antisickling agent possessing dual allosteric and antioxidant effects. Metallomics 9, 1260–1270. 10.1039/c7mt00104e 28770911 PMC5607114

[B33] Kassa T. Whalin J. G. Richards M. P. Alayash A. I. (2021). Caffeic acid: an antioxidant with novel antisickling properties. FEBS Open Bio 11, 3293–3303. 10.1002/2211-5463.13295 PMC8634858 34510823

[B34] Kim Y. Goodman M. D. Jung A. D. Abplanalp W. A. Schuster R. M. Caldwell C. C. (2020). Microparticles from aged packed red blood cell units stimulate pulmonary microthrombus formation via P-selectin. Thromb. Res. 185, 160–166. 10.1016/j.thromres.2019.11.028 31821908 PMC7061313

[B35] Kim-Shapiro D. B. Lee J. Gladwin M. T. (2011). Storage lesion: role of red blood cell breakdown. Transfusion 51, 844–851. 10.1111/j.1537-2995.2011.03100.x 21496045 PMC3080238

[B36] Klein H. G. (2017). The red cell storage lesion(s): of dogs and men. Blood Transfus. 15, 107–111. 10.2450/2017.0306-16 28263166 PMC5336330

[B37] Kottemann M. C. Conti B. A. Lach F. P. Smogorzewska A. (2018). Removal of RTF2 from stalled replisomes promotes maintenance of genome integrity. Mol. Cell. 69, 24–35. 10.1016/j.molcel.2017.11.035 29290612 PMC6467090

[B38] Kuck J. L. Bastarache J. A. Shaver C. M. Fessel J. P. Dikalov S. I. May J. M. (2018). Ascorbic acid attenuates endothelial permeability triggered by cell-free hemoglobin. Biochem. Biophys. Res. Commun. 495, 433–437. 10.1016/j.bbrc.2017.11.058 29129689 PMC5736437

[B39] Leal J. K. F. Adjobo-Hermans M. J. W. Bosman G. J. C. G. M. (2018). Red blood cell homeostasis: mechanisms and effects of microvesicle generation in health and disease. Front. Physiology 9, 703. 10.3389/fphys.2018.00703 PMC6002509 29937736

[B40] Liu T. F. Vachharajani V. Millet P. Bharadwaj M. S. Molina A. J. Mccall C. E. (2015). Sequential actions of SIRT1-RELB-SIRT3 coordinate nuclear-mitochondrial communication during immunometabolic adaptation to acute inflammation and sepsis. J. Biol. Chem. 290, 396–408. 10.1074/jbc.M114.566349 25404738 PMC4281742

[B41] Macdonald R. (1977). Red cell 2,3-diphosphoglycerate and oxygen affinity. Anaesthesia 32, 544–553. 10.1111/j.1365-2044.1977.tb10002.x 327846

[B42] Manalo D. J. Buehler P. W. Baek J. H. Butt O. D'Agnillo F. Alayash A. I. (2008). Acellular haemoglobin attenuates hypoxia-inducible factor-1alpha (HIF-1alpha) and its target genes in haemodiluted rats. Biochem. J. 414, 461–469. 10.1042/BJ20080313 18498252

[B43] Mandal D. Baudin-Creuza V. Bhattacharyya A. Pathak S. Delaunay J. Kundu M. (2003). Caspase 3-mediated proteolysis of the N-terminal cytoplasmic domain of the human erythroid anion exchanger 1 (band 3). J. Biol. Chem. 278, 52551–52558. 10.1074/jbc.M306914200 14570914

[B44] Mteng F. Alayash A. I. (2017). Determination of extinction coefficients of human hemoglobin in various redox states. Anal. Biochem. 521, 11–19. 10.1016/j.ab.2017.01.002 28069451 PMC5303181

[B45] Mohanty J. Nagababu E. Rifkind J. (2014). Red blood cell oxidative stress impairs oxygen delivery and induces red blood cell aging. Front. Physiology 5, 84. 10.3389/fphys.2014.00084 PMC3937982 24616707

[B46] Oh J. Y. Marques M. B. Xu X. Li J. Genschmer K. R. Phillips E. (2023). Different-sized extracellular vesicles derived from stored red blood cells package diverse cargoes and cause distinct cellular effects. Transfusion 63, 586–600. 10.1111/trf.17271 36752125 PMC10033430

[B47] Palha M. S. Legenzov E. A. Lamb D. R. Zimring J. C. Buehler P. W. Kao J. P. Y. (2022). Superoxide measurement as a novel probe of red blood cell storage quality. Blood Transfus. 21, 422–427. 10.2450/2022.0246-22 36580028 PMC10497384

[B48] Pittman R. N. (2011). Regulation of tissue oxygenation. San Rafael (CA): Morgan and Claypool Life Sciences. 21634070

[B49] Rubin O. Canellini G. Delobel J. Lion N. Tissot J. D. (2012). Red blood cell microparticles: clinical relevance. Transfus. Med. Hemother 39, 342–347. 10.1159/000342228 23801926 PMC3678264

[B50] Said A. S. Rogers S. C. Doctor A. (2017). Physiologic impact of circulating RBC microparticles upon blood-vascular interactions. Front. Physiol. 8, 1120. 10.3389/fphys.2017.01120 29379445 PMC5770796

[B51] Seltsam A. (2017). Pathogen inactivation of cellular blood products-an additional safety layer in transfusion medicine. Front. Med. (Lausanne) 4, 219. 10.3389/fmed.2017.00219 29255710 PMC5722787

[B52] Semenza G. L. (2007). Life with oxygen. Science 318, 62–64. 10.1126/science.1147949 17916722

[B53] Song A. Wen A. Q. Wen Y. E. Dzieciatkowska M. Kellems R. E. Juneja H. S. (2022). p97 dysfunction underlies a loss of quality control of damaged membrane proteins and promotes oxidative stress and sickling in sickle cell disease. FASEB J. 36, e22246. 10.1096/fj.202101500RR 35405035

[B54] Storch E. K. Custer B. S. Jacobs M. R. Menitove J. E. Mintz P. D. (2019). Review of current transfusion therapy and blood banking practices. Blood Rev. 38, 100593. 10.1016/j.blre.2019.100593 31405535

[B55] Strader M. B. Jana S. Meng F. Heaven M. R. Shet A. S. Thein S. L. (2020). Post-translational modification as a response to cellular stress induced by hemoglobin oxidation in sickle cell disease. Sci. Rep. 10, 14218. 10.1038/s41598-020-71096-6 32848178 PMC7450072

[B56] Sztiller M. Puchala M. Kowalczyk A. Bartosz G. (2006). The influence of ferrylhemoglobin and methemoglobin on the human erythrocyte membrane. Redox Rep. 11, 263–271. 10.1179/135100006X155012 17207308

[B57] Thiagarajan P. Parker C. J. Prchal J. T. (2021). How do red blood cells die? Front. Physiology 12, 655393. 10.3389/fphys.2021.655393 PMC8006275 33790808

[B58] Tinmouth A. Fergusson D. Yee I. C. Hébert P. C. ABLE Investigators Canadian Critical Care Trials Group (2006). Clinical consequences of red cell storage in the critically ill. Transfusion 46, 2014–2027. 10.1111/j.1537-2995.2006.01026.x 17076859

[B59] Welbourn E. M. Wilson M. T. Yusof A. Metodiev M. V. Cooper C. E. (2017). The mechanism of formation, structure and physiological relevance of covalent hemoglobin attachment to the erythrocyte membrane. Free Radic. Biol. Med. 103, 95–106. 10.1016/j.freeradbiomed.2016.12.024 28007575 PMC5282401

[B60] Wolfe L. C. (1985). The membrane and the lesions of storage in preserved red cells. Transfusion 25, 185–203. 10.1046/j.1537-2995.1985.25385219897.x 3890284

[B61] Yoshida T. Prudent M. D'Alessandro A. (2019). Red blood cell storage lesion: causes and potential clinical consequences. Blood Transfus. 17, 27–52. 10.2450/2019.0217-18 30653459 PMC6343598

[B62] Zhang B. Pan C. Feng C. Yan C. Yu Y. Chen Z. (2022). Role of mitochondrial reactive oxygen species in homeostasis regulation. Redox Rep. 27, 45–52. 10.1080/13510002.2022.2046423 35213291 PMC8890532

